# Trial Designs Likely to Meet Valid Long-Term Alzheimer's Disease Progression Effects: Learning from the Past, Preparing for the Future

**DOI:** 10.4061/2009/949271

**Published:** 2009-12-22

**Authors:** Aaron S. Kemp, George T. Grossberg, Steven J. Romano, Douglas L. Arnold, J. Michael Ryan, Roger Bullock, David L. Streiner

**Affiliations:** ^1^Department of Psychiatry and Human Behavior, UCI Neuropsychiatric Center, Irvine School of Medicine, University of California, 101 the City Drive South, Orange, CA 92868, USA; ^2^Department of Neurology and Psychiatry, Saint Louis University School of Medicine, St. Louis, MO 63104, USA; ^3^Neurosciences, Pain and Inflammation, Pfizer Inc., New York, NY 10017, USA; ^4^Montreal Neurological Institute, McGill University, Montreal, QC, Canada H3A 2B4; ^5^NeuroRx Research, Montreal, QC, Canada H3A 2B3; ^6^Neuroscience, Wyeth Research, Collegeville, PA 19426, USA; ^7^Kingshill Research Centre, Swindon SN3 6BW, UK; ^8^Kunin-Lunenfeld Applied Research Unit, Baycrest Centre, Toronto, ON, Canada M6A 2E1; ^9^Department of Psychiatry, University of Toronto, Toronto, ON, Canada M5T 1R8; ^10^Department of Psychiatry and Behavioural Neurosciences, and Clinical Epidemiology and Biostatistics, Faculty of Health Sciences, McMaster University, Hamilton, ON, Canada L8N 3Z5

## Abstract

The International Society for CNS Clinical Trials and Methodology (ISCTM) held its 4th Annual Autumn Conference in Toronto, Ontario, October 6-7, 2008. The purpose of the present report is to provide an overview of one of the sessions at the conference which focused on the designs and methodologies to be applied in clinical trials of new treatments for Alzheimer's disease (AD) with purported “disease-modifying” effects. The session began with a discussion of how neuroimaging has been applied in multiple sclerosis clinical trials (another condition for which disease modification claims have been achieved). The next two lectures provided a pharmaceutical industry perspective on some of the specific challenges and possible solutions for designing trials to measure disease progression and/or modification. The final lecture provided an academic viewpoint and the closing discussion included additional academic and regulatory perspectives on trial designs, methodologies, and statistical issues relevant to the disease modification concept.

## 1. Introduction

Alzheimer's disease (AD) is a progressive, degenerative disorder of aging that affects cognition, behavior, and overall functioning, and is associated with significant morbidity. Currently available treatments, including cholinesterase inhibitors and an NMDA receptor antagonist, only modestly enhance deficient neurotransmitter systems associated with underlying degenerative processes, and are of limited clinical benefit. As our appreciation of the underlying pathophysiology of AD has increased substantially over the last two decades, new targets for disease intervention have been identified. These include processes associated with the production, modulation, and accumulation of amyloid-beta (A*β*) and tau. These proteins are associated with the neuropathological features of amyloid plaques and neurofibrillary tangles, respectively, and are hypothesized to be involved in disease processes that contribute to progressive synaptic dysfunction, neurodegeneration, and cell death.

Based on this expanding understanding, new molecular entities are being developed that may disrupt a cascade of events hypothesized to lead to progressive neurodegeneration. As these treatments impact on more primary processes, they could modify disease progression and offer clinical benefits beyond shorter-term symptomatic improvement. However, there are significant challenges associated with designing studies that will demonstrate disease modification and support regulatory approvals for this novel indication. The purpose of this session was to examine some of these challenges by first looking at what can be gleaned from other conditions for which disease modification claims have been achieved and then by identifying some of the specific hurdles and possible solutions for designing trials in patients with Alzheimer's disease. Industry, academic, and regulatory agency viewpoints were discussed, followed by robust audience participation.

The session topic was introduced by George T. Grossberg (St. Louis University School of Medicine) who stated that a key question to examine, as recently posed to him by colleague Ravi Anand (Anand Pharma Consulting) was “Is it the molecule or is it the methodology?” He noted that this question has particular relevance to the session topic of trial designs, as the evaluation of innovative new molecular entities (NMEs) for treating AD is fundamentally bounded by the adequacy of the methodologies employed to measure their putative effects on disease progression. Dr. Grossberg then introduced session cochair, Steven J. Romano (Pfizer, Inc.), who in turn introduced the first lecturer, Douglas Arnold (Montreal Neurological Institute and NeuroRx Research). 

## 2. Lecture 1: Douglas Arnold

The topic of Dr. Arnold's lecture was “The use of magnetic resonance imaging (MRI) to measure degeneration and progression: Lessons learned from multiple sclerosis (MS).” Dr. Arnold, an expert in MRI and MS, stated that there are lessons pertaining to the use of neuroimaging in the drug development process for MS which might inform future studies of AD progression. Dr. Arnold provided three key points that he hoped his lecture would convey: (1) though MRI may adequately demonstrate disease pathology, there may be times when findings do not conform to expectations and may necessitate a reconsideration of certain concepts of pathogenesis; (2) MRI findings may dissociate from clinical presentation or course and may raise questions regarding current concepts of pathogenesis; (3) MRI may be a predictive tool to aid in determining diagnostic specificity, prognosis, and potential responsiveness to treatment. 

Gadolinium-enhanced (Gd+) MRI of focal white-matter inflammatory lesions has had a profound impact on drug development in the field of MS by providing sensitive markers of disease activity, and the accumulation of white matter lesion volume also has had an important role in quantifying the “burden” of disease and the effect of disease modifying drugs. The quantification of new, acute lesions is more statistically powerful than the related clinical outcome of MS relapses, can help predict conversion to clinically definite MS in patients who present with a first attack suggestive of MS, and has been partially validated as a surrogate measure of the disease-modifying effects of treatment. The implications for AD trials include the potential for MRI to provide biomarkers that may be more sensitive to disease progression than clinical measures, to identify preclinical indicators of conversion to AD, and to be more responsive to treatment than conventional measures of change. 

Advanced MRI measurements, such as whole brain volume change, magnetization transfer ratio (MTR), T2 relaxation times, and N-acetylaspartate density (a marker of neuronal integrity measured in vivo using magnetic resonance spectroscopy), also may be used to provide novel insights into the mechanisms subserving pathogenesis. For example, these measures have been utilized to determine whether brain volume changes following an experimental treatment for MS, which involved immunoablation with chemotherapy and autologous stem cell transplantation, were more likely the result of actual tissue loss (atrophy) or the resolution of inflammatory edema (pseudoatrophy). As decreased brain volume was found to correlate with MTR (a marker of myelin content) and not with T2 relaxation time (a marker of water content), it was determined that accelerated brain atrophy following chemotherapy was most likely attributable to an actual loss of tissue. Such findings have been crucial for dissociating treatment-related effects from disease progression in clinical trials for MS and provide a lesson for how such MRI measurements could yield equally valuable revelations concerning the mechanisms purported to subserve the pathogenesis and progression of AD and observed responses to therapy.

 Another important lesson learned in the MS field has been that the apparent dissociation of MRI findings from clinical presentation does not necessarily negate their potential use as markers of treatment efficacy. Indeed, involvement of “clinically silent” regions of the brain and compensatory mechanisms such as cerebral reorganization may weaken the associations between MRI pathology and clinical expression but careful examination of these dissociations may further enhance understanding of the overall course of the illness under investigation and provide convergent evidence of treatment effects. For example, a recent meta-analysis of all evaluable trials presented by Maria Pia Sormani (San Raffaele Scientific Institute, Milan, Italy) and colleagues at the World Congress on Treatment and Research of Multiple Sclerosis indicated that despite a poor correlation between Gd-enhancing lesions and clinical relapses on an individual basis, the efficacy of MS treatments in clinical trials as measured by MRI and by clinical outcomes were, nonetheless, highly correlated. Similarly, the use of MRI-based measurements in AD clinical trials offers the potential to provide measures of disease progression that are more powerful than clinical measures. As well, MRI can increase our understanding of the mechanisms underlying pathogenesis, response to treatment, and the relations of such processes to clinical manifestations. In summary, MRI in MS has reduced the sample size and duration of phase II trials and has provided important biological support for disease modification associated with clinical efficacy in phase III trials. It has potential as a surrogate outcome, at the “trial-level,” but it is still not a qualified surrogate after 15 years of use in MS clinical trials.

## 3. Lecture 2: Steven J. Romano

The next lecture at the session was entitled “An industry viewpoint on clinical trial designs for disease modification in AD: Challenges” and was presented by Steven J. Romano (Pfizer, Inc.). Dr. Romano summarized the issues to be addressed in order to advance the development of innovative disease modifying treatments for AD as follows: (1) disease etiology: though much is known about the pathophysiology of AD, there is still much that needs to be more fully elucidated, such as the primary etiological “trigger(s)” and where to best direct interventions; (2) disease modification concepts: still evolving, leaving many questions such as whether (and to what degree) a robust, durable effect is “good enough” to qualify as “modification”; (3) regulatory guidance: also still evolving, particularly with regards to what may constitute a disease-modifying effect; (4) biomarkers: though promising, none has been sufficiently validated to the extent that may be required to gain regulatory acceptance as a surrogate endpoint; and (5) trial design: among the issues that must be resolved are methodological and statistical issues concerning the stratification of the population to be studied, the appropriate endpoints to be employed, the duration and general design of the trials, and statistical power assumptions to address differing rates of change that may be expected with a purported “disease-modifying” agent. 

**Figure 1:**
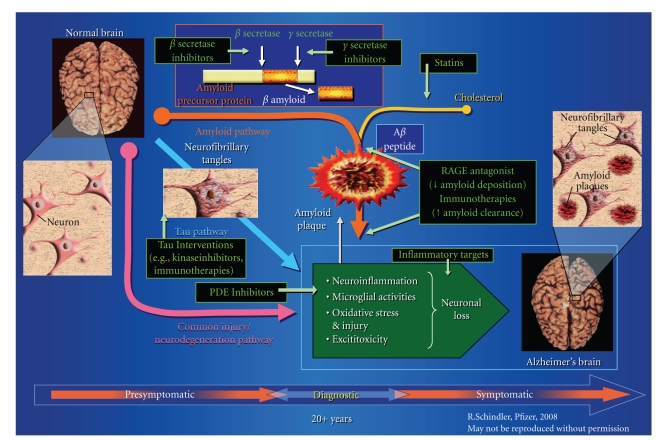
Conceptual overview of the primary etiological mechanisms believed to underlie the development of Alzheimer's disease. Black boxes provide potential targets for interventions at various stages of the disease pathway. Image used with the permission of its creator, Rachel Schindler (Pfizer, Inc.).

Dr. Romano noted that the “good news” is that several central pathophysiological mechanisms believed to contribute to AD have been identified and provided a brief overview of what is currently known (summarized in Figure 1). This expanding appreciation of AD pathophysiology has allowed for the identification of many promising targets for a variety of putative disease-modifying treatment approaches. These include the inhibition or modulation of *γ*- and *β*-secretase, tau, or certain phosphodiesterases (PDE), as well as novel methods to decrease amyloid deposition or promote amyloid clearance. While this situation provides a target-rich arena for potential pharmacological investigation, many fundamental questions remain unanswered, particularly regarding where best to intervene and whether a single point or multiple points of intervention are needed to effect clinically meaningful change. Despite our purported understanding of AD pathophysiology, “Proof of Mechanism” has yet to be translated into a robust “Proof of Concept” in an appropriately powered randomized clinical trial, particularly for any pharmacologic intervention based on the amyloid hypothesis. 

**Figure 2:**
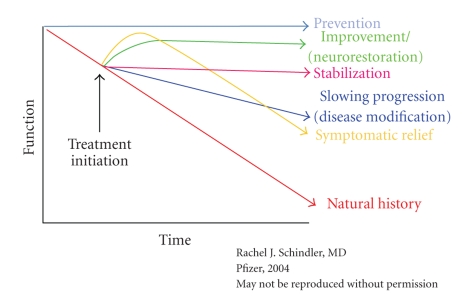
Hypothetical trajectories depicting the changes in level of functioning across time that would be expected due to the natural progression of AD, or with other conceptualized interventions that would produce a symptomatic relief, a disease modifying effect, stabilization, improvement (neurorestoration), or prevention, respectively. Image used with the permission of its creator, Rachel Schindler (Pfizer, Inc.).

Dr. Romano then provided a brief review of the concept of “disease modification,” including a hypothetical graphic depiction (Figure 2) of how this concept contrasts with changes in level of functioning across time that would be expected due to the natural progression of AD, or with other conceptualized interventions that would produce symptomatic relief or (more optimistically) stabilization, improvement (neurorestoration), or prevention. Among the outstanding issues pertaining to the disease modification concept that still need resolution are questions about how best to define and quantify modification; how long it would take to observe a treatment difference; how large an effect might be expected; whether “minor” changes that take 18–24 months to confirm are relevant; at what point in the illness would intervention be most effective; and how to drive academic and regulatory consensus for earlier intervention in populations “at risk” when considering the logistical challenges of identifying such individuals or the ethical dilemma of whether the risks associated with treatment exposure are justifiable.

Regulatory agencies serve a critically important role in resolving many of these outstanding issues, although guidance is still evolving. For example, although biological evidence to support a claim of disease modification is required, there are to date no regulatory agency-endorsed biomarkers of such. Though neuroimaging alone may not suffice, some combination of clinical outcomes and structural MRI (once appropriately validated) may soon overcome this regulatory hurdle. Another challenge is the divergence between US and European regulatory agencies regarding the acceptance of comparisons of slopes (rates of change) using standard parallel-arm studies. While such designs are generally acceptable for European agencies, the US Food and Drug Administration (FDA) appears to prefer other designs (e.g., randomized start/withdrawal) for demonstrating disease modification. 

An additional issue which presents a challenge to the pharmaceutical industry is that the necessary validation of the purported biomarkers for AD progression may end up being confirmed in rather than prior to long-term treatment trials. Although the benefits to be gained from such are readily evident, this does present a considerable risk to the study sponsor, particularly since so many questions still exist concerning whether the characteristics of subpopulations may influence the behavior of specific biomarkers, if the utility of a given biomarker will vary across disease stages, and (perhaps most importantly) whether changes in the biomarker will translate into clinically meaningful benefits. Many of these questions may soon be answered by large-scale, multi-site, longitudinal studies such as the Alzheimer's Disease Neuroimaging Initiative (ADNI), which is supported by joint funding from the pharmaceutical industry, private philanthropic organizations (the Alzheimer's Association and the Alzheimer's Drug Discovery Foundation), and several federal agencies within the US Department of Health and Human Services (including the FDA, the National Institute on Aging, and the National Institute for Biomedical Imaging and Bioengineering). 

The final set of challenges broached by Dr. Romano was the specific trial design issues that must be addressed before launching additional large-scale treatment trials. Among the many that will require further discussion among academic, regulatory, and industry representatives to resolve are methodological questions such as whether to use a parallel-arm or randomized start/withdrawal designs (addressed further below); whether the population to be studied should be earlier-stage AD patients and whether it should be stratified by either severity or genotype; what would be the required duration of the trial to demonstrate disease modification within a minimum timeframe; which clinical, neuroimaging, or biochemical measures are most appropriate to employ as either efficacy or safety endpoints and how are these to be standardized in multinational trials; and what statistical methods or assumptions would be required to sufficiently power a trial to detect meaningful differences between the natural disease progression and an unknown degree of change associated with a novel intervention. In closing, Dr. Romano summarized the overall implications of the challenges he raised by stating that demonstration of disease modification will invariably require large, long, costly trials; and that the significant risks yet to be resolved include: unprecedented mechanisms of action with as yet no clear proof of concept, limitations of current translational models (most models support proof of mechanism but do not provide true disease models), a lack of sufficient validation of biomarkers, the fact that regulatory guidance is not yet harmonized or “written in stone,” and a huge unknown regarding the potential value proposition of these new treatments to “payers” or policy-makers within the healthcare industry. 

## 4. Lecture 3: J. Michael Ryan

The next lecture at the session was entitled “An industry viewpoint on clinical trials designed for demonstrating disease progression in AD: Current approaches” and was presented by J. Michael Ryan (Wyeth Research). Dr. Ryan began by offering the perspective that the industry's apparent perseveration on the notion of “disease modification” versus symptomatic therapies is not merely a matter of “chasing label claims” but instead reflects the current consensus that supporting failing neurotransmitter systems is “just not good enough.” By comparison, a treatment which offers disease-modifying effects would actually increase in terms of clinical benefits across time by altering the natural progression of the disease. 

Emerging markers of disease progression (e.g., cerebrospinal fluid analytes, various MRI measures, or more sensitive cognitive measures) may actually provide better “signal strength” than traditional measures of clinical expression, which may be “imperfect reporters of disease state” despite having been canonized over time as efficacy endpoints. In a recent publication by Vellas et al. [1] from the European AD Consortium (EADC), the consensus of experts and regulators on the appropriate endpoints to be applied in various “symptomatic,” “prevention,” or “disease-modifying” treatment trials actually shows surprisingly little variation in the tools recommended to gauge efficacy or track progression, regardless of trial-type or the severity of the population studied. While more innovation has been evident in the area of cognitive assessment, more still may be needed to improve the sensitivity of other measures of clinical expression (e.g., activities of daily living assessments). 

 Dr. Ryan turned next to a brief discussion of the oft-suggested, yet rarely-executed, randomized start and withdrawal (RS/RW) trial designs. Dr. Ryan presented figures from Leber's [2] influential publication suggesting the utility of these trial designs for distinguishing disease-modifying from symptomatic treatment effects. A variation on a cross-over design, RS/RW designs employ two sequential treatment segments in which either the initiation or the withdrawal of the experimental treatment is delayed for a randomly selected subset of subjects. If the experimental group “catches up” with the active comparison group in the RS design or declines to the level of the placebo group in the RW design then a symptomatic effect is assumed. If, however, the experimental group shows sustained benefits relative to the comparison groups then a disease-modifying effect is assumed. To explain why these designs have rarely been employed on long-term AD trials, Dr. Ryan mentioned several issues of concern which have made the industry unwilling to fully support their usage. Specifically, he noted that while treatment effects will likely differ across severity stages of AD, the two-period dichotomy of RS/RW designs may accentuate the impact of treatment-by-time interactions. Furthermore, the fact that the optimal duration of the delayed-withdrawal or staggered-start segments is currently unknown; empirical questions regarding how to establish the relationships between complex pharmacokinetic, pharmacodynamic, and clinical effects; the concern that the negative impact of dropouts would be amplified; and the difficulties of modeling nonlinear changes in clinical outcomes, were all mentioned as issues that have hampered the adoption of the RS/RW designs by industry. 

Reiterating some of the concerns raised earlier by Dr. Romano regarding the critical need to validate biomarkers, Dr. Ryan suggested the leading candidates that may eventually achieve surrogate status following replication across multiple compounds and trials are volumetric MRI, cerebrospinal levels of A*β* or tau, positron emission tomography (PET), or ratios/combinations of these measures. For a particular compound, these measures may be used now in the “learn” phase of development to justify dose selection or in “confirm” phase trials as supportive data if treatment benefit is demonstrated on the primary clinical outcomes. The European Medicines Agency (EMEA) has stated that “ideally, proof of a disease-modifying effect would require demonstration of clinically relevant changes in key symptoms of the dementia syndrome and in addition supportive evidence for a change in the underlying disease process based on biological markers.” However, the necessary steps for the qualification or validation of proposed biomarkers have not been explicitly addressed by either the EMEA or FDA. For now, Dr. Ryan noted that the EMEA suggests a two-step approach to the disease modification claim: (1) “delay of disability” based solely on clinical outcomes and (2) “full claim for disease modification” if a convincing biomarker package supports the clinical outcomes. 

 Dr. Ryan then provided a brief overview of the relevance of apolipoprotein E (APOE) and its variant subtypes (particularly *ε*4). According to a recent report by Jiang et al. [3], APOE is believed to stimulate the degradation of A*β* in the brain, with the *ε*4 subtype being the least effective variant, thereby leading to greater A*β* burden (including greater vascular deposition) among patients with the *ε*4 genotype. Carriers of the APOE *ε*4 genotype have an increased risk of developing AD, may present an altered course of disease progression among AD patients, and may also show differential responses to treatment compared with patients with other variants of APOE. For these reasons, Dr. Ryan cautioned that future trials should be designed to identify (and possibly stratify) participants on the basis of whether they are carriers of the APOE *ε*4 genotype (which have generally comprised approximately 60% of trial participants). 

In closing, Dr. Ryan addressed methodological and statistical issues concerning the importance of minimizing missing data and summarized the key topics that he felt were critical for designing AD trials to demonstrate disease modifying effects. Citing an additional consensus report from the EADC (Vellas et al. [4]), Dr. Ryan summarized their recommendations (and his own) as follows: the target population should include patients with early or mild to moderate AD (and stratification on the basis of APOE genotype should be considered); the study design should be a randomized, parallel, two-arm, placebo-controlled trial of at least 18 months in duration (or longer); proposed statistical analyses would include a slope analysis (when not precluded by nonlinearity in the data); primary endpoints should be clinically relevant and include measures of cognitive functions, functional status, neuropsychiatric symptoms, and cost-effectiveness; and secondary endpoints may include biomarkers (biological and neuroimaging), but such are not currently recommended as surrogate measures of primary outcome. 

## 5. Lecture 4: Roger Bullock

The final lecture at the session was entitled “An academic viewpoint on clinical trial designs for disease modification in AD: Challenges” and was presented by Roger Bullock (Kingshill Research Centre). Among the specific challenges that Dr. Bullock mentioned would require further clarification were conceptual problems with: the disease, the science, the investigational products, the methodology, and the drug development process. For each of these conceptual challenges, Dr. Bullock provided a brief summary of how the perspectives of academics, clinicians, and regulatory bodies pertain to (or differ with) the current industrial imperatives to develop new treatments for AD. 

Regarding the conceptual problems with the disease itself, Dr. Bullock mentioned that there are uncertainties regarding the natural course of the amyloid deposition process, particularly in light of recent evidence that suggests amyloid levels may actually *decline* across time. Accordingly, Dr. Bullock asserted that if the clinical impairments continue to increase in severity without an apparent increase in brain amyloid then certainly there are other processes at work that must be more fully understood. This also relates to the concerns with the science, which Dr. Bullock also mentioned, particularly regarding the interpretation of findings derived from the use of amyloid-imaging biomarkers such as the Pittsburgh Imaging compound-B (PIB). Dr. Bullock also echoed comments by the other presenters by stating that currently available biomarkers still require considerable validation to evaluate their relevance to clinical expression, disease progression, and treatment responsiveness; that the elaborate modes of action of certain proposed therapies have not yet resulted in a robust “proof of concept;” and that adequate translational (animal) models are still lacking. 

Dr. Bullock also raised conceptual problems with the investigational products by questioning the notion that “one magic pill” could be developed to treat all of the problems associated with AD. Although regulatory agencies may not currently license products for the treatment of specific symptoms, it may be useful to match modes of action with specific clinical targets, thus narrowing the focus of treatment development with the hope that such therapies could potentially be used in combination. As an example, Dr. Bullock mentioned that “apathy” can be among the most bothersome symptoms for caregivers and treating this specific symptom (among others) may be key to producing “real-world” functional outcomes. 

Dr. Bullock then turned to a discussion of specific methodological issues such as whether it is appropriate to employ the same scales and designs to measure disease-modifying agents as have been used to assess symptomatic treatment effects. While such methods were designed primarily to monitor increasing disabilities, it would be nice to have designs or measures which would be more sensitive to *improvements* in functioning, particularly when focusing on the early stages of AD, during which cognitive impairments are relatively minimal. Dr. Bullock noted that he favors the use of measures to assess functional abilities, including activities of daily livings (ADLs) or dependency scales (about which he noted colleague Yaakov Stern may soon release seminal findings). Dr. Bullock also recommended that more emphasis be placed on measures of cognition which may relate more directly to functional abilities, particularly assessments that may be more sensitive to the relations between “executive control” processes and ADLs. Specifically, he suggested that treatments which improve “executive control” over global brain functions (not just “executive functions” as typically measured by “frontal-lobe,” performance-type tests) may be mirrored by greater improvements in ADLs, than might a treatment which only affects discrete memory processes. Dr. Bullock also noted that he favors “person-centered” clinical measures such as the Goal-Attainment Scaling (GAS) developed by Rockwood et al. [5, 6]. 

In closing, Dr. Bullock reviewed some concerns with the drug development process, in general, and posed some suggestions for moving forward. Specifically, he noted that in the haste to develop new products too many “positive” phase II safety studies have resulted in “negative” phase III efficacy studies. He stated that, financial imperatives aside, some of the blame for this may reside with “unimaginative” advisory boards or the circular exchange of dialogue between academic, industry, and regulatory representatives based on fixed ideas and vague expectations. As such, he challenged each to be more vocal in their expression of dissenting viewpoints when such are held and have not been voiced, and encouraged a “regrouping” of what has already been learned about AD so that better “more focused” clinical targets may be revealed. He suggested that perhaps there are too many questions to be answered at once, and that focusing on answering the smaller questions first may produce better results. Finally, Dr. Bullock closed with a quotation from T.S. Elliot: “Only those who risk going too far can possibly know how far they can really go”.

## 6. Panel Discussion with David L. Streiner

Following the final lecture, Drs. Grossberg, Arnold, Romano, Ryan, and Bullock were joined on stage by an additional academic discussant, David L. Streiner, who is the Research Director at the Baycrest Centre, Professor of Psychiatry at the University of Toronto, Professor of Psychiatry and Behavioral Neurosciences, and Emeritus Professor of Clinical Epidemiology and Biostatistics at McMaster University School of Medicine. Before opening the floor for a panel discussion, Dr. Grossberg asked Dr. Streiner to provide his own perspective on the topics raised in the session. 

Dr. Streiner noted that there are many parallels with the field of schizophrenia research regarding the heterogeneity of the population that is currently being defined by a constellation of symptoms that are collectively referred to as one disease entity, namely AD. Although many clinically distinct diagnostic variations of dementia exist (e.g., Lewy-body dementia, or frontal-lobe dementia), there may yet be many other subtypes of AD that would need to be distinguished by differing etiologies to make meaningful progress in the field. Dr. Streiner also mentioned that he had detected disagreement among the presenters (although not explicitly stated) concerning whether to focus primarily on biomarkers or functional measures of behavior as efficacy endpoints. As a statement of his perspective on this issue, Dr. Streiner suggested that AD patients are not institutionalized because of abnormal MRI or laboratory results but because the family cannot cope with the behavioral or functional impairments. Further, as there is currently a dearth of evidence showing any causal relations between the purported biomarkers and functional behaviors, he believes that biomarkers should remain secondary measures for clinical trials. 

The panel then fielded questions from the audience, leading to many intriguing discussions. Among the many knowledgeable session attendees who posed questions, Lon S. Schneider (University of Southern California, Keck School of Medicine) noted that the dynamic interplay between industry and academia has provided a particular definition of “mild to moderate AD” that presents a large market opportunity, but that more focus should be directed toward defining sources of patient heterogeneity and distinguishing subtypes of dementia that may prove even more relevant for both industrial and academic research purposes. Regarding this topic, Cristina Sampaio (Faculdade de Medicina de Lisboa) mentioned that although it may currently be impossible to accurately identify certain subpopulations (particularly, “at-risk” or “pre-dementia” cases), this would not preclude regulatory agencies from considering trials directed at certain readily identifiable subpopulations (e.g., “familial-type” AD, or APOE *ε*4 carriers) or other biomarker-defined subtypes, once they have been properly validated. Thus, Dr. Sampaio (an academic regulatory reviewer, herself) challenged the notion that regulatory agencies have narrowed the focus of drug development to the “mild to moderate AD” definition and encouraged industry to move beyond this particular paradigm. Further, Dr. Sampaio also disagreed that regulatory agencies would not consider treatments targeting specific symptoms, though she did note that establishing the case for a particular symptom such as “apathy” would be difficult considering the common comorbid presentation of depression. As an example, she noted that cognition in schizophrenia has been established as a valid target indication, but only after thorough validation of the construct as a core characteristic of the illness and the identification of valid instruments to quantify changes in this particular symptom. 

Additional session attendees who raised interesting topics of discussion included, Andrew C. Leon (Weill Medical College of Cornell University), Ravi Anand (Anand Pharma Consulting), and Suzanne Hendrix (Pentara Corporation), who each stimulated detailed discussions regarding statistical issues, such as the implications of both attrition and “competing risks” on modeling slopes, the subtle distinctions between “missing at random” and “missing not at random” when using mixed-effects models, and the need for sensitivity analyses to test whether the model can account for attrition with either outcomes captured prior to drop-out or other post-randomization predictors. Steven G. Potkin (University of California, Irvine School of Medicine) mentioned that the dissociation of biomarkers (e.g., neuroimaging measures) from clinical expression may not be a troubling finding, since such measures may actually provide more sensitivity to potential disease-modifying effects earlier in the course of treatment than may clinical measures. Finally, Johannes Streffer (Johnson & Johnson Pharmaceutical Research) noted that it is critically important to acknowledge that biomarkers must be validated with a specific purpose in mind, as a valid biomarker for diagnostic purposes may or may not have appropriate utility to also track progression or disease modification and vice versa.
